# Prevalence of intestinal parasites in street dwellers attending a shelter in Cali, Colombia

**DOI:** 10.7705/biomedica.7269

**Published:** 2024-11-06

**Authors:** Jorge Iván Zapata-Valencia, Diana Maritza Jurado-Orejuela, Ofelia Flórez-Echeverry, Érica Marcela Aristizábal-Giraldo, Jhonathan León Gallego-Franco, María Camila Yolanda Ramírez-Uribe, Jemina Rentería-Molina, Alejandra Sandoval-Villareal, Yermaín Ulabarri-Valencia, Juan Carlos Zambrano-Camelo

**Affiliations:** 1 Escuela de Bacteriología y Laboratorio Clínico, Facultad de Salud, Universidad del Valle, Cali, Colombia1 Universidad del Valle Escuela de Bacteriología y Laboratorio Clínico Facultad de Salud Universidad del Valle Cali Colombia

**Keywords:** Intestinal parasites, poverty, shelter, Colombia, homeless, parásitos intestinales, pobreza, refugio, Colombia, habitante de calle

## Abstract

**Introduction.:**

Intestinal parasitic infections represent a public health problem, especially among vulnerable populations. There are few studies on the prevalence and determinants of intestinal parasites infections in street dwellers, who may experience significant health and socioeconomic implications. Understanding the prevalence and associated factors of intestinal parasites in this population is crucial for targeted interventions to mitigate the spread of these infections.

**Objective.:**

To determine the prevalence of intestinal parasites in street dwellers attending a shelter in Cali, Colombia.

**Materials and methods.:**

We selected 66 participants who met the inclusion criteria. We collected serial stool samples for laboratory evaluation and sociodemographic data, and information on their hygiene habits and addictions.

**Results.:**

Seventy-six percent of the participants had intestinal parasites or commensals, with 30% presenting monoparasitism, 46% polyparasitism, and 20% eosinophilia. *Blastocystis* spp. was the most common organism (68.18%), followed by *Endolimax nana* (34.85%) and *Entamoeba coli* (18.18%). The most common pathogens were the *Entamoeba histolytica/E. dispar/E. moshkovskii* complex (10.61%) and hookworms (9.09%). We evaluated prevalence-related determinants.

**Conclusions.:**

Intestinal parasitism is a health problem among street dwellers in Cali. A serial examination is recommended for diagnosing intestinal parasitic infection, especially in cases of low parasite loads. Campaigns should be established to reduce the prevalence of these parasites in populations at risk of complications.

## Resumen

**Introducción.:**

Las enteroparasitosis representan un importante problema de salud pública, especialmente entre poblaciones vulnerables. Hay pocos estudios sobre la prevalencia y los factores determinantes relacionados con la presencia de enteroparásitos en habitantes de calle, quienes pueden sufrir complicaciones de salud e implicaciones socioeconómicas significativas. Por lo tanto, comprender la prevalencia y los factores asociados con los enteroparásitos en esta población, es crucial para desarrollar intervenciones dirigidas y mitigar la propagación de estas infecciones.

**Objetivo.:**

Determinar la prevalencia de enteroparásitos en habitantes de calle que acuden a un hogar de paso en Cali, Colombia.

**Materiales y métodos.:**

Se seleccionaron 66 participantes que cumplían con los criterios de inclusión y se recolectaron muestras seriadas de materia fecal para su evaluación en el laboratorio. Se recopiló información sociodemográfica y datos sobre hábitos de higiene y adicciones de los participantes.

**Resultados.:**

El 76 % de los participantes presentó parásitos o comensales intestinales: el 30 % tenía monoparasitismo, el 46 %, poliparasitismo, y el 20%, eosinofilia. *Blastocystis* spp. fue el organismo más frecuente (68,18 %), seguido por *Endolimax nana* (34,85 %) y *Entamoeba coli* (18,18 %). Entre los agentes patógenos, los más comunes fueron el complejo *Entamoeba histolytica/E. dispar/E. moshkovskii* (10,61 %) y las uncinarias (9,09 %). Se evaluaron los factores determinantes relacionados con estas prevalencias.

**Conclusiones.:**

El parasitismo intestinal sigue siendo un problema importante en grupos vulnerables, como los habitantes de calle en Cali. Se recomiendan exámenes seriados para el diagnóstico de las parasitosis intestinales, especialmente en casos de bajas cargas parasitarias. Es necesario establecer campañas para disminuir la prevalencia de estos parásitos en poblaciones con riesgo de complicaciones.

Street dwellers in Colombia are individuals who reside and live on the streets, whether temporarily or permanently, regardless of their sex, age, or race ^1^^,^^2^. They typically have low socioeconomic status, inadequate personal and environmental hygiene, and limited access to clean drinking water. They face challenges in accessing healthcare services and often do not receive adequate medical treatment for their acute or chronic illnesses due to a lack of knowledge, substandard healthcare quality, stigmatization by healthcare providers, or insufficient financial resources, among other barriers hindering comprehensive healthcare ^3^^-^^7^. These characteristics render them a vulnerable population deserving attention from the government and society. Given their condition, they can serve as carriers or reservoirs of infectious diseases, posing a risk to the wider population interacting with them and representing a public health concern in numerous countries ^8^.

**Figure 1:**
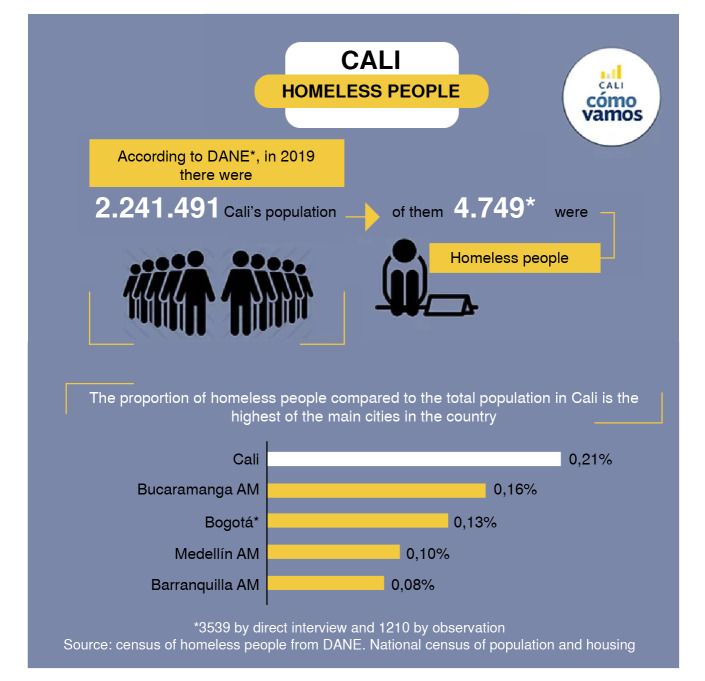
Proportion of homeless people from Cali's total population compared to other cities in the country

Source: modified from https://www.calicomovamos.org.co/habitantes-de-calle?lightbox=dataItem-k96699kw

Some studies have estimated that the global homeless population is 500 million ^9^. In 2005, a sectoral census conducted in Cali identified 3,620 individuals in this situation, 86.2% men and 13.8% women. Half of them found residing or sleeping in the neighborhoods of Alameda, Bretaña, Belalcázar, Sucre, and Obrero in District 9, among others; and El Calvario, San Pascual, San Juan Bosco, San Pedro, and San Nicolás in District 3 ^10^. A subsequent census in 2019 showed a 31.18% increase, with 4,749 homeless individuals (1,129 more than in 2005). At that time, Cali had the highest proportion of homeless people among the major capitals of the country (figure 1) ^11^.

* DANE: Departamento Administrativo Nacional de Estadística de Colombia

Several studies have shown that environmental factors such as residing in tropical climates with low altitude, poor sanitary conditions (including inadequate personal and environmental hygiene), limited access to clean drinking water, and deficient disposal of human waste influence the prevalence of intestinal parasites, along with economic factors such as low family and population income, and social conditions like overcrowding and overpopulation ^12^^-^^14^.

Street dwellers' health status is understudied in many aspects ^15^. For example, research on intestinal parasites among adult and child street dwellers is scarce. Studies in Venezuela ^16^, Brazil ^17^, Ethiopia ^18^^,^^19^, the Philippines ^20^, and Perú ^15^ have revealed up to 71.8% prevalence of intestinal parasites. The prevalence rates for individual commensal or pathogenic helminths and unicellular species vary between 1.1% and 58.2%. *Trichuris trichiura* and *Ascaris lumbricoides* are frequently reported helminths, while *Blastocysts* spp. and *Giardia duodenalis* are prevalent unicellular organisms. Certain intestinal helminths and protozoa frequencies suggest inadequate fecal elimination practices, indicative of poor water quality intended for human consumption in some countries ^21^^,^^22^.

We found no studies on this topic in Colombia; therefore, there is a lack of information on the prevalence or incidence of intestinal parasitic infections among this population. This epidemiological information is important for designing appropriate control strategies for this highly vulnerable population. Here, we determined the prevalence of enteroparasites in street dwellers attending a shelter in Cali.

## Materials and methods

We recruited 107 individuals of both sexes and ages between 22 and 87 years, and we enrolled 66 who fulfilled the inclusion criteria: voluntary participation, signing the informed consent, providing survey information and at least one stool sample for analysis, and not having received antiparasitic treatment for at least two months before the study.

After the informed consent procedure, each participant answered a survey about their health status and sociodemographic information. They were provided with containers and instructed on how to collect stool samples. Once collected, the samples were transported under refrigeration to the *Laboratorio de Parasitología* of the *Escuela de Bacteriología y Laboratorio Clínico* at the *Universidad del Valle.*

We used direct examination with saline, Lugol's iodine solution, zinc sulfate flotation, and acid-fast staining to search for parasites. Each participant and the shelter medical staff received a copy of the direct wet smear analysis report for inclusion in their medical records. The medical staff prescribed antiparasitic treatments and managed the medical care accordingly.

### Ethical considerations

We contacted shelter staff and potential participants and informed them about the purpose of the study. The project was approved by the *Comité de Ética para la Investigación en Salud* of the *Facultad de Salud* at the *Universidad del Valle* (approval certificate #018-018).

## Results

According to the sociodemographic data (table 1), 89% of the study population was male, and the remaining percentage was female. Participants had moved to Cali for family reasons, escaping violence, seeking job opportunities, and other motivations. Of the 66 participants, 37 (56.06%) were originally from Cali, 22 (33.33%) had been living in the city for five or more years, 6 (9.10%) for less than five years, and one (1.51%) participant did not provide information about his place of birth. Drug abuse was the main reason (34/66; 51.51%) leading the participants to become street dwellers. Living arrangements varied, with some residing in rooms and elsewhere.

**Table 1:** Sociodemographic and hygiene characteristics of the homeless people participating in the study (n = 66)

Characteristic		n	%
Gender	Males	59	89.40
	Females	7	10.60
Age group (years)	20 - 39	16	24.24
	40 - 59	33	50.00
	60 - 69	9	13.63
	> 70	5	7.58
	No information	3	4.55
Education level	Primary school (complete or incomplete)	18	27.27
	Secondary school (complete or incomplete)	39	59.09
	College (complete or incomplete)	5	7.58
	No education	3	4.55
	No information	1	1.51
Ethnicity	Mestizo	27	40.90
	White	15	22.72
	Afro-descendant	11	16.66
	Mulatto	7	10.60
	Native	3	4.55
	No information	3	4.55
Circumstances leading participants to become street dwellers	Drugs	34	51.51
	Death of family members/no family	9	13.63
	Family issues	7	10.60
	Job's lost/economic issues	3	4.55
	Own decision	2	3.03
	Others (sentimental or emotional issues, family abuse,	9	13.63
	alcohol, lawsuits, loss of housing, no other option)		
	No information	2	3.03
Time spent living on the street (years)	5 or more	48	72.72
	3 - 4	8	11.12
	1 - 2	4	6.06
	1 or less	4	6.06
	No information	2	3.03
Place of origin	Cali	38	56.10
	Other Valle del Cauca municipalities	12	18.18
	Caldas	3	4.55
	Risaralda	3	4.55
	Antioquia	2	3.03
	Atlántico	2	3.03
	Bogotá	2	3.03
	Cauca		1.51
	Chocó		1.51
	Santander		1.51
	Quindío		1.51
	No information		1.51
Reasons for relocating to Cali	No information	38	57.57
	Family	12	18.18
	Violence	7	10.60
	Job opportunities	5	7.58
	Other	4	6.06
	Study	0	0.00
Main meals per week	Daily	52	78.79
	Once per week	0	0.00
	Twice per week	5	7.58
	Four times per week	4	6.06
	Five times per week	2	3.03
	No information	3	4.54
Having a partner	Yes	15	22.73
	No	51	77.27
Shelter attendance frequency	Daily	56	84.85
	Six times per week	3	4.54
	Five times per week	3	4.54
	Four times per week	2	3.03
	Thrice per week	1	1.52
	No information	1	1.52
Water source	Tap water	60	90.90
	Other (bottled water, rivers, public fountains)	5	7.58
	No information	1	1.52
Washing hands before eating	Yes	55	83.33
	No	11	16.67
Washing hands after going to the toilet	Yes	58	87.88
	No	8	12.12

Education levels ranged from incomplete or complete high school, incomplete or complete primary school, higher education, and no formal education. Ethnicity encompassed mestizo, white, afro-descendant, and mulatto; the marital status included single, divorced, common-law marriage, and married individuals. Most reported having no partners (51/66; 77.27%) and some having one partner (15/66; 22.73%) (table 1).

Regarding hygiene and sanitation practices, the majority reported consuming a minimum of three meals per day (49/66; 74.24%), some two meals per day (16/66; 24.24%), and one participant did not provide information on this (1/66; 1.52%). As for shelter attendance frequency, most participants reported daily attendance (56/66; 84.85%), nine attended six, five, four, or three times per week (9/66; 13.63%), and one did not provide information on this (1/66; 1.52%) (table 1).

The majority (56/66; 84.85%) reported never consuming spoiled food, some (9/66; 13.63%) admitted to doing so, resorting to scavenging food from streets or discarded by restaurants, or asking for food on the streets, and one participant (1/66; 1.52%) did not provide information on this. In terms of raw food consumption, half of the participants reported abstaining, while others (32/66; 48.48%) said they consumed raw foods, primarily vegetables, greens, and fruits, and one (1/66; 1.52%) participant did not provide information on this; one participant mentioned consuming raw rice and potatoes, and another one preferred partially cooked meat (medium well).

As for water consumption, the majority (60/66; 90.9%) drank tap water, five participants (7.58%) consumed water from various sources, including bottled water, rivers, public fountains, or a combination of these; some drank a mix of tap and bottled water, or tap water and water from public fountains, and one (1/66; 1.52%) participant did not provide information on this (table 1).

Regarding their health status, some participants reported having at least one disease (23/66; 34.84%), with 15 (65.22%) under medical supervision or taking prescribed medication. A minority followed a specific diet (2/66; 3.03%), and some took food supplements (12/66; 21.21%). More than half of the participants smoked (35/66; 53.03%), some consumed alcohol (19/66; 28.28%), and over half used hallucinogens (40/66; 60.61%). When asked about changes in weight over the past year, most (45/66; 68.18%) reported they had experienced them, and one-third (22/66; 33.33%) had cramps and diarrhea in the weeks leading up to the study.

In terms of hygiene habits, most respondents reported washing their hands before eating (55/66; 83.33%) and after going to the toilet (58/66; 87.88%), some acknowledged defecating outdoors (22/66; 33.33%), and more than half urinated in the open air (36/66; 54.54%) (table 1). Almost all reported wearing shoes (64/66; 96.97%), some engaged in activities involving contact with dirt or grass (23/66; 34.85%), and a few indicated they owned pets (3/66; 4.54%).

Among the 66 participants, 49 (74.24%) contributed three serial fecal samples, 6 (9.1%) provided two, and 11 (16.66%) provided only one sample, totaling 170 stool samples. The overall prevalence of intestinal parasites or commensals was 75.75%, with 50 out of 66 participants testing positive for any of the intestinal microorganisms; a subset of 16 participants (24.24%) had no parasites or commensals (table 2).

**Table 2:** Prevalence of intestinal parasites and commensals in street dwellers living in Cali (n = 66)

Parasites/commensals	I**nfected**	
	n	%
*Blastocystis* spp.	45	68.18
*Endolimax nana*	23	34.85
*Entamoeba coli*	12	18.18
*Entamoeba histolytica/E. dispar/E. moshkovskii* complex	7	10.61
Hookworms	6	9.09
*Entamoeba hartmanni*	4	6.06
*Giardia duodenalis*	1	1.52
None	16	24.24

In descending order of occurrence, the parasites identified in this cohort were *Blastocystis* spp., *End. nana, Ent. coli,* the *Ent. histolytica/E. dispar/E. moshkovskii*complex, hookworms, *E. hartmanni,* and *Giardia duodenalis.* No intestinal apicomplexans were detected using acid-fast staining (table 2).

Regarding associated parasitic infections, monoparasitism was found in 20 of the 66 (30.30%) of the participants, polyparasitism in 30 (45.45%), with 15 (22.73%) having two parasites, 12 (18.18%) three parasites, and 3 (4.54%) four or more parasites. Of those with parasitic infections, 10/50 (20%) exhibited eosinophilia, with 9/10 (90%) presenting *Blastocystis* spp. as a sole parasite or in coinfections with hookworms.

According to Fisher's exact test, sex (p = 0.67) and age (p = 0.96) were not related to the presence of parasites. As for the association between colic or diarrhea in the two weeks before the study, there was a statistical significance between the two variables (p = 0.03) according to the same test. In the bivariate analysis of risk factors and intestinal parasitism among the 66 participants, we found that 50 individuals had parasites irrespective of the water source they utilized (table 3). Regarding cramps and diarrhea in the weeks before the study related to the presence of parasites, we found that all 22 individuals with cramps had intestinal parasites (table 4).

**Table 3:** Presence of parasites according to water sources used by homeless people attending a shelter in Cali, Colombia (n = 66)

Water source	Without parasites	With parasites	Total
Bottled	--	2	2
Tap	1	59	60
Rivers	--	2	2
Other	--	1	1
No information	--	1	1
Total	1	65	66

**Table 4:** Presence of colic and diarrhea prior to the parasitism study in street dwellers attending a shelter in Cali, Colombia (n = 66)

Cramps or diarrhea in the last two weeks	Without	With	Total
	parasites	parasites	
Yes	0	22	22
No	1	41	42
No information	0	2	2
Total	1	65	66

## Discussion

Several studies have indicated that various circumstances can lead individuals to become street dwellers, including experiences of sexual, psychological, and physical abuse, and being ostracized by their families due to drug or alcohol addiction issues ^5^^,^^23^. This was also the case with the participants in our study, besides the emotional struggles, childhood abuse within their families, legal troubles, and homelessness due to loss of housing.

Homeless individuals are vulnerable and experience chronic health issues and barriers to accessing healthcare. Also, they have inadequate nutrition and exhibit high rates of alcohol and substance abuse ^4^^,^^5^^,^^23^. Intestinal parasitism has been extensively studied in various populations worldwide, including adults, elderly populations, children, and school-aged individuals, but not among street dwellers despite their heightened vulnerability. This type of study is particularly scarce in Colombia and, worldwide, only a few have focused on adult and children street dwellers, primarily in some African countries ^13^^,^^16^^-^^18^^,^^24^^-^^26^.

Our study found various parasites in this population. However, unlike research conducted on street dwellers in other countries ^16^^,^^24^, infections caused by single-celled microorganisms (parasites and commensals) were predominant, with only six (9.09%) cases of hookworm infections detected (9.09%). In a study conducted with 80 alcoholic homeless adults from Ciudad Bolívar, Venezuela, other stool tests were used, revealing a parasitism rate of 61.3% ^16^. *Blastocystis* spp. and *End. nana* exhibited the highest prevalences among parasites and commensals, with rates of 33.8% and 21.3%, respectively, albeit in lower proportions compared to our research. The Venezuelan study reported low frequencies of soil-transmitted helminths, *Strongyloides stercoralis* being the most prevalent (12.5%). *Ent. coli* (8.8%) and hookworms (3.8%) were also identified among other intestinal organisms but in lower proportions than those observed in our study.

In Rio de Janeiro, Brazil, a study to assess the prevalence of intestinal helminth infections among homeless individuals ^17^ involved 82 participants with ages ranging from 9 months to 46 years. The overall prevalence of parasites was 63.4%, with *A. lumbricoides* being the most common helminth at 48.8%, followed by *T. trichiura* at 32.9% and hookworms at 8.5%. Monoparasitism was observed in 37.9% of participants and polyparasitism in 25.5%, with the most frequent combination being *A. lumbricoides* and *T. trichiura* (16.0%). In contrast, our study did not detect any infections with these two parasites, and hookworm infections were present in a higher proportion (9.09%). Besides, monoparasitism was less common (30%), and polyparasitism was more prevalent in our findings (46%).

In another study among street dwellers in Dessie, Ethiopia ^24^, enteroparasite prevalence was 43.9%. Six parasites, including the *Ent. histolytica/E. dispar/E. moshkovskii complex* (9.8%) and *G. duodenalis* (5.3%), were identified, with no hookworm infections detected. Monoinfections accounted for 35.8% and infections by two parasites 8.13%, but unlike our findings, there were no reports of multiple infections by three or more parasites. The average age of the participants in this study was 22.85 years, and most of them had lived on the streets for more than a year. Similar to our results, poverty (19.5%) and addictions (35%) were cited as the main reasons for becoming homeless, and lack of hand washing may also be associated with enteroparasite infection (45.3%).

In Gondar, Ethiopia, a study conducted on street dwellers (n = 404) to investigate enteroparasites and HIV ^25^ found that among the primary reasons for participants ending up on the streets were rural-to-urban migration driven by poverty (52.7%), divorce (16.8%), family bereavement (28.0%), and influences of addiction and peer pressure (2.5%), similar to the participants in our study. Additionally, 6.9% tested positive for HIV, and 67.6% were positive for intestinal parasites, with 27.7% exhibiting multiple infections involving two or more parasites. The study identified eight types of helminths and two protozoa, with the highest prevalences observed for *A. lumbricoides* (36.8%), hookworms (26.2%), and *T. trichiura* (21.0%). The prevalence of *G. duodenalis* and the *Ent. histolytica/E. dispar/E. moshkovskii* complex was 6.9% and 3.5%, respectively. It is worth noting that while there are similarities in the parasites identified, the prevalences may not be directly comparable due to the larger sample size in the Gondar study compared to ours.

Another study of 355 street dwellers in Addis Ababa, Ethiopia, found a general prevalence of 71.8% for intestinal parasitic infections with one or more species ^9^. The predominant parasites were helminths, with *A. lumbricoides* at 34.9%, *T. trichiura* at 22.8%, and *Taenia* spp. at 17.5%. Among protozoa, *G. duodenalis* was reported at 9.6%, and the *Ent. histolytica/E. dispar/E. moshkovskii* complex at 8.2%. Hookworms were found in 1.7% of participants. Comparatively, the parasitism rates for amoebas and hookworms in this study were lower than ours, yet a higher prevalence of *G. duodenalis* was noted.

Another study conducted in Jimma, Ethiopia, to assess the prevalence of enteroparasites among street dwellers (n = 116) revealed that 86.9% of participants harbored one or more enteroparasites, with 54.3% of them hosting two or three parasites ^18^.The most prevalent were *A. lumbricoides* (65.5%) and *T. trichiura* (44.8%), while hookworms, *G. duodenalis,* and the *Ent. histolytica/E. dispar/E. moshkovskii* complex had prevalence rates of 9.5, 3.4, and 4.3%, respectively. Notably, *G. duodenalis* prevalence was lower in our study, that of the *Ent. histolytica/E. dispar/E. moshkovskii* complex was higher, and hookworm prevalence was similar.

A systematic review and meta-analysis ^13^ conducted on 17 published studies examining enteroparasites in street dwellers and prisoners revealed a general prevalence of 43.68% for both populations (n = 4,544 participants), with a higher prevalence (68.39%) observed among 1,494 street dwellers, although lower than in our study. The analysis also identified *A. lumbricoides* (14.07%), *Ent. histolytica* (7.83%), and *G. duodenalis* (7.49%) as the most common parasites in both groups, while hookworms exhibited a prevalence of 2.70%. These figures contrast our findings, in which the prevalences of *Ent. histolytica* and hookworms were higher, but that of *G. duodenalis* was lower.

Other studies have centered on intestinal parasites in street children. Although our study focused on adults, it is worth comparing these results with ours. A survey conducted among orphaned and homeless children in Argentina (n = 396) showed an intestinal parasitism prevalence (including commensals and pathogens) of 84.8% ^26^. The study highlighted *G. duodenalis* (23%), *Ent. coli* (45.5%), *Blastocystis* spp. (44.4%), and *End. nana* (34.6%) high prevalences, among others. Multiparasitism was evident in 64% of the children, with 24% infected by two species, 18% by three species, and 22% by four or more species. Monoparasitism was observed in 21% of the participants, while 15% showed no evidence of parasitic infection.

A similar study was conducted among street children in Perú (n = 258), who were cared for in orphanages across three districts of Lima ^15^. Each participant provided a sample that was then analyzed for intestinal parasites and commensals. Results revealed that 66.3% of the participants tested positive for intestinal parasites and/or commensals, with 30.6% hosting pathogenic species; 31.0% of the samples contained a single parasite, and 35.3% showed polyparasitism. *Entamoeba coli* emerged as the most prevalent intestinal commensal protozoan (41.9%), followed by *G. duodenalis* as the most common pathogenic protozoan (17.1%); both species had higher prevalences compared to those in our study. *Ascaris lumbricoides* (62.8%), *T. trichiura* (4.7%), hookworms (1.2%), and *Cyclospora cayetanensis* (3.5%) were also identified among other parasites and commensals such as *End. nana* (32.2%), *lodamoeba butschlii* (0.4%), and *Chilomastix mesnili* (11.6%) were prevalent as well among the participants.

A study conducted in Manila, Philippines, among children who were cared for in institutions (n = 204) and from street communities (n = 80) ^20^ found a general prevalence of 62% for enteroparasites ^20^. Children from institutions had 59.8% of infections attributed to helminths and 44.1% to protozoans, whereas 52.5% of the children from street communities had helminths and 48.8% protozoans. Polyparasitism was observed in 34.2% of cases. The prevalence of hookworms was 7%, *Blastocystis* spp. accounted for 40.7%, *G. duodenalis* was detected in 11.6% of cases, and *Ent. histolytica/E. dispar/E. moshkovskii* complex was 2.9%.

Another study in Jimma, Ethiopia, involving street children (n = 312) collected a single sample per participant and showed that 66.7% of them harbored intestinal parasites, with 15.9% exhibiting polyparasitism ^19^. The predominant parasites were *A. lumbricoides* (58.2%) and *T. trichiura* (13.5%). Hookworms were detected in 7.7% of the participants, *G. duodenalis* (10.6%) emerged as the most prevalent protozoan, and the *Ent. histolytica/E. dispar/E. moshkovskii* complex had a prevalence of 3.4%.

The infection patterns observed in street children align closely with those identified among adults in our study. Similar to our findings, *Blastocystis* spp., *End. nana,* and *Ent. coli* exhibited the highest prevalences among the organisms detected. Polyparasitism (46%) falls within an intermediate range compared to the rates observed in children, possibly influenced by factors such as the age of the participants and their sheltered living conditions.

Differences in enteroparasite distribution between our study population and other studies in similar populations are evident, with a higher prevalence of protozoa compared to helminths. Various factors may contribute to these variations, including differences in socioeconomic and climatological conditions across regions, the timing of sampling, sample sizes, age demographics, number of fecal samples analyzed per participant, methodologies used for detecting enteroparasites, presence of underlying diseases, and participants' food sources ^23^. Unlike many other studies where participants obtained food from hotels or leftovers, our participants received food at a shelter, potentially reducing their exposure to certain parasites. Besides, they received primary medical and dental care at the shelter, which may further reduce the prevalence of intestinal parasites. However, we observed high frequencies in our study. The organisms found could have been acquired through contaminated water or foods, particularly raw vegetables prone to be contaminated during production, transportation, sale, or handling.

We underscore the significance of two parasites identified in our study. Firstly, *Blastocysts* spp. emerged as the most prevalent organism (68.18%), a noteworthy finding considering that Ethiopian studies with similar populations typically did not report the presence of this parasite, whose pathogenic role remains controversial despite being recognized for over a century. While some studies associate *Blastocystis* spp. specific subtypes with gastrointestinal symptoms (such as abdominal pain, diarrhea, and vomiting, besides urticarial and peripheral and tissue eosinophilia), others fail to establish conclusive evidence of these associations ^27^^-^^36^.

The presence of *Blastocystis* spp. in our study might explain why 33% of the participants experienced abdominal pain, and 20% of the infected individuals exhibited eosinophilia. *Blastocystis* spp. was found either in monoinfection or coinfection with other organisms, including hookworms. The prevalence of *Blastocystis* spp. found in our study (68.18%) was higher than that of the National Intestinal Parasitism Survey for the Andean Region (60.5%) among the street dwellers evaluated. However, it is relevant to mention that while *Blastocystis* spp. prevalence was higher, handwashing frequency was lower among participants in our study (83%) compared to those in the national survey (88%) ^37^, which primarily involved school children.

On the contrary, our study stands out for identifying hookworms as the sole helminth present, albeit with minimal parasite loads, as evidenced by the sparse detection of eggs in direct examinations or solely in concentration or serial tests ^38^. Given the longevity of these worms ^39^, it is conceivable that participants were already infected upon their arrival in Cali and went undiagnosed, or they contracted the infection through skin contact with contaminated soil during recycling work or bathing in the Cali river, as reported by some participants. Comparatively, hookworm prevalence in our study (9.09%) exceeds that of studies conducted in Ciudad Bolívar, Venezuela (3.8%) ^16^ and is slightly higher than that in Rio de Janeiro, Brazil (8.2%) ^17^.

Although eosinophilia is a common finding in helminthiasis, only one hookworm-positive participant in our study exhibited the condition, likely due to the low parasite load. Eosinophils are known to accumulate at the tissue level in response to larvae presence (L_3_e), with peripheral blood eosinophilia observed when larvae levels are sufficient ^40^.

The higher prevalence of hookworms in our study compared to the Andean Region's national survey (1.6%) ^37^ may be attributed to the socio-economic conditions prevalent among our participants, characterized by poverty and extreme poverty, and their increased likelihood of skin contact with contaminated soil, as noted by some participants.

The prevalence of intestinal parasites in our study highlights the frequent exposure to stool-contaminated food and water. Despite participants' reports of wearing shoes, some individuals afflicted with hookworms admitted to occasionally bathing in the Cali river, suggesting it may be a primary source of infection.

The insights provided by studies on intestinal parasitosis across various geographical, climatic, and social contexts will enable health authorities to develop and implement more effective control strategies against these parasites in our context, ensuring the inclusion of all susceptible populations, particularly the most vulnerable and marginalized.
